# Effects of group metacognitive training (MCT) on mental capacity and functioning in patients with psychosis in a secure forensic psychiatric hospital: a prospective-cohort waiting list controlled study

**DOI:** 10.1186/1756-0500-5-302

**Published:** 2012-06-18

**Authors:** Marie Naughton, Andrea Nulty, Zareena Abidin, Mary Davoren, Sarah O’Dwyer, Harry G Kennedy

**Affiliations:** 1 National Forensic Mental Health Service, Central Mental Hospital, Dundrum, Dublin, 14, Ireland; 2 Department of Psychiatry, Trinity College, Dublin, Ireland

## Abstract

**Background:**

Metacognitive Training (MCT) is a manualised cognitive intervention for psychosis aimed at transferring knowledge of cognitive biases and providing corrective experiences. The aim of MCT is to facilitate symptom reduction and protect against relapse. In a naturalistic audit of clinical effectiveness we examined what effect group MCT has on mental capacity, symptoms of psychosis and global function in patients with a psychotic illness, when compared with a waiting list comparison group.

**Methods:**

Of 93 patients detained in a forensic mental health hospital under both forensic and civil mental health legislation, 19 were assessed as suitable for MCT and 11 commenced. These were compared with 8 waiting list patients also deemed suitable for group MCT who did not receive it in the study timeframe. The PANSS, GAF, MacArthur Competence Assessment Tool- Treatment (MacCAT-T) and MacArthur Competence Assessment Tool-Fitness to Plead (MacCAT-FP) were recorded at baseline and repeated after group MCT or following treatment as usual in the waiting list group.

**Results:**

When baseline functioning was accounted for, patients that attended MCT improved in capacity to consent to treatment as assessed by the MacCAT-T (p = 0.019). The more sessions attended, the greater the improvements in capacity to consent to treatment, mainly due to improvement in MacCAT-T understanding (p = 0.014) and reasoning . The GAF score improved in patients who attended the MCT group when compared to the waiting list group (p = 0.038) but there were no changes in PANSS scores.

**Conclusion:**

Measures of functional mental capacity and global function can be used as outcome measures for MCT. MCT can be used successfully even in psychotic patients detained in a forensic setting. The restoration of elements of decision making capacity such as understanding and reasoning may be a hither-to unrecognised advantage of such treatment. Because pharmacotherapy can be optimised and there is likely to be enough time to complete the course, there are clear opportunities to benefit from such treatment programmes in forensic settings.

## Background

Although pharmacotherapy remains the main treatment for schizophrenia and other psychotic illnesses, patients are often dissatisfied with this and discontinue or change their prescribed medication [1]. Moreover, approximately 20-30 % of patients are resistant to antipsychotic medication [2]. Cognitive Behavioural Therapy (CBT) is an evidence-based adjunct to medication in the treatment of schizophrenia. Evidence has developed from case studies, randomised controlled trials and meta-analyses [3,4] confirming the effectiveness of CBT for persistent positive and negative symptoms of schizophrenia, usually with small to medium effect sizes. The understanding of cognitive processes and biases in schizophrenia has expanded significantly in recent years [5]. New approaches to the treatment of schizophrenia have evolved from research on cognitive biases and distortions evident in psychosis, including Metacognitive Therapy (MCT) [6]. In forensic settings, patients are often detained because of impaired functional mental capacities for example if found unfit to stand trial. These functional cognitive deficits are likely to have the same basis in cognitive biases and distortions and may benefit from the same treatments.

In view of the empirical findings [7] suggesting deficits of metacognition (thinking about one’s thinking, reflecting upon one’s cognitive processes) in patients with schizophrenia, metacognitive training (MCT) is increasingly adopted as an adjunct treatment approach [8,9]. A number of problematic thinking styles or cognitive biases reported in schizophrenia are related to the formation and maintenance of positive symptoms of schizophrenia, particularly delusions. Among these biases are attributional biases, the jumping to conclusion bias, bias against disconfirmatory evidence, deficits in theory of mind, over confidence in memory errors and depressive cognitive patterns [9-11]. MCT aims to sharpen patients' awareness of a variety of cognitive biases that are implicated in the formation and maintenance of schizophrenic positive symptoms especially delusions, and to replace these biases with more adaptive cognitive strategies. Studies confirm the feasibility [9] and lend preliminary support to the efficacy [8,9,12] of the intervention. There is a growing body of literature on the use of MCT in psychosis, however to the authors’ knowledge there is no evidence of its use patients for with psychotic illness in the forensic population.

Aghotor et al. [9] showed an improvement in all subscales of the Positive and Negative Syndrome Scale (PANSS) in psychotic patients relative to an active control group. Positive symptoms attenuated with a medium effect size of d = 0.43. In addition, results showed a reduced bias towards jumping to conclusions for MCT patients (d = 0.31). However, none of these effects reached statistical significance. The authors suggested that small sample size (n = 30 randomly assigned to MCT or an active control group) and similarities between the programmes (the control intervention partly involved metacognitive judgements) with regard to content may have masked stronger differences. While these studies begin to provide some modest evidence for the efficacy of MCT in reducing positive symptoms of psychosis, how this translates into meaningful improvements in general functioning and functional mental capacities for decision making remains to be shown.

In forensic practice there is a growing awareness of the need to assess functional mental capacity to make decisions in relation to the exercise of legal rights for example fitness to stand trial or fitness to give or withhold consent to treatment. For the most part in clinical practice if a situation causes a clinician to examine competency to make a decision, he or she will use unstructured professional judgement to assess functional mental capacity. However research instruments employing structured professional judgements have recently improved the reliability of such assessments [13,14]. In a comparative empirical study [15] it was shown that substantially more patients with schizophrenia were classified as impaired using the objective MacArthur Competence Assessment Tool (MacCAT-T) than by clinical assessment. In a forensic setting separate functional mental capacities such as consent to treatment and fitness to stand trial are not independent of each other and when measured, overlap and correlate substantially while measurements of different functional mental capacities correlate with global function and correlate inversely with scores for severity of psychosis [16]. Mental capacity for functions essential to the exercise of legal rights is commonly impaired amongst patients with psychosis in a forensic setting and the more information to be dealt with when making an informed decision to consent, the more likely it is that a person will fall short of a test of decision making capacity [17].

In this study we hypothesized that (i) patients with a primary psychotic illness who undertook a course of MCT would show improved measurements on positive and negative symptoms of schizophrenia (PANSS), on capacity to consent to treatment, and on global functioning. We hypothesized that (ii) capacity assessment regarding fitness to plead would remain unchanged as the functional components of this assessment are not incorporated into MCT.

## Methods

### Study Design

This was a prospective naturalistic cohort study, instigated as part of the clinical audit service evaluation process at the National Forensic Mental Health Service for Ireland.

This is not a randomized controlled trial. It is doubtful that such a study could be carried out in a detained population of patients, many of whom lack capacity to give or withhold consent for treatment or for such a trial. In the population of patients in a forensic secure hospital, there is a necessity to deliver effective interventions to patients who may be more ill than those in other settings. We have previously shown [16,17] that in such patients, with very similar mean scores for PANSS positive, negative and general scales, GAF and MacCAT-T, 25 % of those who appeared to consent to a research assessment on capacity to consent to treatment were actually unable to make a decision, increasing to 37.5 % when given extra information. Others [18] have found as many as 60 % of psychiatric in-patients lacked sufficient capacity to give consent to treatment. A series of previous studies in the same population had demonstrated that a substantial proportion of the patients did not have the capacity to consent to treatment [16,17], arguably a less complex process than a randomised controlled trial (RCT) where misunderstandings can be common [19,20]. Clinical trials are not permitted for patients formally detained under section 70 of the Mental Health Act 2001 for Ireland [21], p152-153], a higher standard than that defined in international conventions such as the Orviedo convention, article 17 [22]. The exclusion of some groups from exacting protocols is itself a source of bias in the published literature and may lead to over-estimation of the effectiveness of an intervention when applied to those with the most severe forms of illness [23].

This demonstrates the difficulty of carrying out an RCT in such a population and the importance of focusing on effective ways to improve mental capacity when auditing the effectiveness of treatment interventions.

There is also sufficient evidence of efficacy of MCT as a means of improving symptoms to make an RCT difficult to justify in such a group, even though there are no studies using mental capacity as an outcome measure that we know of. Resource limitations meant we could not treat all those who might benefit at once. This offered the opportunity of a waiting list comparison group who appeared to have no differences from the treatment group that would cause bias. Therapeutic effectiveness needs to be constantly evaluated with clinical audits to ensure treatment programmes are achieving their intended goals.

The study was approved by the National Forensic Mental Health Service Research and Audit Ethics and Effectiveness Committee in the Central Mental Hospital, Dublin. All patients referred for Metacognitive Therapy (MCT) were given an explanation of the nature and purpose of the treatment and its evaluation for audit purposes. All patients consented to the treatment and evaluation. As the hospital clinical programme was to deliver the intervention to those who would assent to it after a standard consent procedure, the committee approved the assessment protocol as good clinical practice to ensure that effectiveness was known as part of the continuing audit of the effectiveness of treatment programmes.

### Setting

At the time of the study (September 2009 – June 2010), the National Forensic Mental Health Service for Ireland had 93 secure in-patient beds, including eight for women, at the Central Mental Hospital. The Central Mental Hospital is the only hospital in Ireland that admits patients remanded or sentenced to prison, or found not guilty by reason of insanity so that the patients included here are typical of those detained in other jurisdictions under both forensic and civil mental health legislation [24]. The male wards (units) are organised into a coherent pathway through care from high to medium to low security and pre-discharge [25]. Patients who are assessed as at lower risk than on admission (usually in association with partial or complete remission of symptoms of psychosis) progress through the hospital to less secure units where there is opportunity for psychological treatments to address physical and mental health, substance misuse problems, problem behaviours and social, occupational and family issues [26]. These are addressed in the form of treatment programmes such as metacognitive training (MCT), enhanced thinking skills, dialectic behavioural therapy, anger management and education regarding healthily lifestyles and relationships.

### Intervention: metacognitive training (MCT)

According to its authors [6], the metacognitive training programme (MCT) is based on two fundamental principles. The first is knowledge translation. Cognitive biases are explained comprehensively to patients and illustrated by multiple examples. Jargon is avoided. The second principle is demonstration of the negative consequences of cognitive biases. Exercises targeting each bias and which demonstrate the fallibility of human cognition are discussed within the group. Personal examples of these biases are expressed by MCT participants and discussion of ways to counter them serves to provide corrective experiences in a relaxed and supportive atmosphere, yielding obvious advantages over mere didactic information giving. Patients are taught to recognise and counter the biases that are important in schizophrenia, thus allowing them to arrive at more appropriate inferences and avoiding automatic “cognitive traps” [9,27].

Metacognitive training (MCT) was delivered twice a week for 8 weeks between October and December 2009 by two healthcare specialists, a psychiatrist and a clinical nurse specialist, both qualified as recommended in the handbook, p5 [28] who then trained in delivering MCT by reading the modules and handbook and by a period of joint preparation supervised by the senior clinician (AN). The programme [29] and handbook were made available to the service free of charge from the designers of the training programme [28]. The programme consists of 8 modules over 16 sessions (2 sessions per module) consisting of pdf-converted PowerPoint slides. Each module familiarised participants with the respective topic (e.g. jumping to conclusions) and multiple exercises were administered, aimed at challenging the functionality of biased thinking styles and providing corrective experiences. The main objective of the training is to raise the participants’ awareness of these cognitive distortions and to prompt them to critically reflect on, complement and alter their current repertoire of problem solving skills. The modules are concluded with learning goals and a case example to show participants how cognitive biases can escalate to psychotic symptoms. While highly structured, the layout and presentation is visually stimulating, information is simplified with no medical jargon and the exercises generated lively discussions and differing views were exchanged. Informal feedback following the groups was that they were enjoyed by participants [9,30].

### Variables

For abnormalities of mental state, the Positive and Negative Syndrome Scale (PANSS) [31] was used which yielded scores for positive and negative symptoms, general symptoms and a total score. We also used the Global Assessment of Functioning Scale (GAF) [32] as a measure of general functional competence. These were rated by their treating psychiatrists as part of periodic routine assessments and collated in September 2009 and again in March 2010.

The most extensively researched and validated instruments for the measurement of functional mental capacities emphasize the capacities to understand relevant information, to reason about the task in hand, and to appreciate the relevance of the information and reasoning to one’s self [33]. The MacArthur Competence Assessment Tool- Treatment (MacCAT-T) [34,35] measures understanding, reasoning and appreciation in relation to proposed treatment. In addition it records whether the patient was able to make a choice or not. To rate the MacCAT-T for this study, the same procedure was followed as described by Rutledge et al. [16]. All participants were offered information first about their illness then about two oral antipsychotic medications, olanzapine and risperidone. The information was read from a prepared script derived from the data sheets and summary of product characteristics published by the regulatory authority [36]. Participants were given 2 benefits and 2 possible side effects of each medication along with 2 benefits and 2 possible side effects of no medication. Participants were scored in the areas of understanding, reasoning and appreciation in relation to their selected option.

The MacArthur Competence Assessment Tool-Fitness to Plead (MacCAT-FP) has been validated for use in the UK [37] based on a tool developed in the USA [38]. A vignette is read to the person being tested and questions are asked so that the researcher can rate the three mental capacities, understanding, reasoning and appreciation relevant to the patient’s impending trial.

Participants were interviewed and scored for the MacCAT-T and MacCAT-FP by MN, trained in the use of the measurement tools in September 2009 prior to treatment and March 2010 after treatment. The post-treatment assessments could not be done completely blind to treatment status.

The DUNDRUM-1 triage security scale is a recently designed prospective, validated, structured professional judgement instrument designed to assess need for various levels of therapeutic security and appropriateness for admission to a secure forensic psychiatric hospital [24,39]. These were carried out blinded to treatment status. The HCR-20 [40] is a widely used, validated structured professional judgment instrument for identifying risk factors for violence, to be used in planning treatment and care.

### Participants

**Figure 1:**
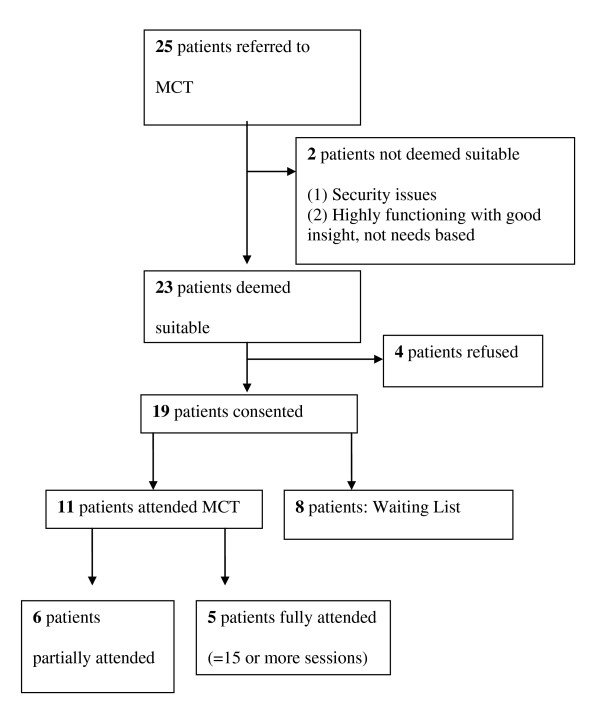
CONSORT flow diagram.

Twenty five in-patients in the Central Mental Hospital were referred by their multidisciplinary team for MCT because they had incomplete responses to anti-psychotic medication (Figure 1). Two patients were not deemed suitable; one for security issues and the second as the patient was deemed to be highly functioning with good insight. Four patients refused to participate. Nineteen patients consented to treatment with MCT and to participate in the assessments.

All nineteen patients met the DSM-IV-TR criteria [32] for a psychotic disorder (schizophrenia = 15, schizoaffective disorder = 3, major depressive disorder with psychotic features = 1).

The nineteen were allocated to a treatment group or a waiting list on a 'first come first served' (chronological) basis; 11 patients attended the first MCT programme between October and December 2009 and 8 patients were waiting list controls. All the patients were male and the mean age was 36.7 years (SD 10.59). All spoke adequate English. Of the 11 who attended MCT, five fully attended (attended fifteen to sixteen of sixteen sessions) and six partially attended (fourteen or less sessions).

Table 1 shows baseline demographic data on all patients that attended MCT compared to waiting list controls. There were no differences between the MCT group and the waiting list comparison group in age (F = 0.02 df = 17 p > 0.9), length of stay (F = 0.71 p > 0.4), diagnosis (Fisher’s exact test = 3.2, p > 0.2), and whether on Clozapine or not (*X*^2^ = 2.4 df = 1 p > 0.1). Table 2 shows assessments for the PANSS, GAF, MacCAT-T total, MacCAT-FP total and Dundrum 1 Assessment, indicating that there were no differences in these variables between the two groups at baseline. Table 3 shows that there were no differences at baseline between the MCT and comparison groups in lack of insight, negative attitudes or unresponsiveness to treatment, though there were significant differences in substance misuse problems, exposure to destabilisers and stress.

**Table 1:** Participants’ Demographic Data at Baseline means (standard deviations)

	**Metacognitive Training group n = 11**	**Waiting list group n = 8**	**Statistics**
**Age (years)**	37.5 (10.6)	35.62 (11.2)	F = 0.02, df = 17, p > 0.9
**Length of Stay (months)**	44 (47.8)	48 (55.5.)	F = 0.9, df = 17, p > 0.4
**Diagnosis (n)**			
Schizophrenia	7	8	Fisher's exact = 3.2, p > 0.2
Schizoaffective Disorder	3	0	
Major Depression with Psychotic Features	1	0	
**Medications (n)**			
Clozapine	8	3	*X*^2^ = 2.4, df = 1, p > 0.1.
Other	3	5	

**Table 2:** Baseline Assessments for the Positive and Negative Symptoms of Schizophrenia, Global Assessment of Function and DUNDRUM-1 triage security score

	**Metacognitive Training group n = 11Mean(SD)**	**Waiting list group n = 8Mean(SD)**	**Significance (ANOVA, F/p)**
**PANNS pos**	11.4 (3.7)	14.0 (6.3)	1.3/>0.2
**PANNS neg**	17.7 (6.7)	17.5 (4.7)	0.0/>0.9
**PANNS gen**	31.7 (8.6)	27.0 (6.6)	1.7/>0.2
**PANNS tot**	60.7 (15.2)	58.8 (14.9)	0.8/>0.7
**GAF**	50.6 (9.5)	54.6 (8.8)	0.9/>0.3
**Mac Cat – T**	12.9 (4.1)	11.8 (4.3)	0.3/>0.5
**(Total)**			
**Mac Cat – FP**	25.5 (6.5)	23.9 (7.5)	0.3/>0.6
**(Total)**			
**Dundrum 1 Assessment**	30.6 (4.9)	29.1 (4.2)	0.9/>0.3

**Table 3:** Risk factors present at baseline taken from the HCR-20

	MCT group N = 11Mean (SD)	Waiting list group N = 8Mean (SD)	Mann–Whitney-UZ/p
H1 - previous violence	2.00(0.00)	2.00(0.00)	0.0/>0.9
H2 - young age at first violence	1.00(0.47)	1.25(0.46)	−1.1/>0.2
H3 - relationship instability	1.50(0.71)	1.50(0.76)	0.05/>0.9
H4 - employment problems	1.30(0.82)	1.25(0.89)	−0.1/>0.9
H5 - substance misuse problems	1.40(0.69)	2.00(0.00)	−2.3/0.023
H6 - major mental illness	2.00(0.00)	2.00(0.00)	0.0/>0.9
H7 - psychopathy	0.00(0.00)	0.00(0.00)	0.0/>0.9
H8 - early maladjustment	1.00(0.82)	1.13(0.99)	−0.3/>0.7
H9 - personality disorder	0.20(0.42)	0.50(0.53)	−1.3/>0.1
H10 - prior supervision failure	1.60(0.69)	1.25(0.89)	−0.9/>0.3
C1 - lack of insight	1.30(0.48)	1.63(0.52)	−1.3/>0.1
C2 - negative attitudes	0.50(0.71)	0.75(0.89)	−0.6/>0.5
C3 - active symptoms	1.25(0.89)	1.25(0.89)	−1.7/>0.05
C4 - impulsivity	0.13(0.35)	0.13(0.35)	−0.2/>0.8
C5 - unresponsive to treatment	1.13(0.83)	1.13(0.83)	−1.02/>0.3
R1 - plans lack feasability	0.50(0.76)	0.50(0.76)	−1.7/>0.05
R2 - exposure to destabilisers	0.38(0.52)	0.38(0.52)	−2.1/0.039
R3 - lack of personal support	0.38(0.74)	0.38(0.74)	−0.2/>0.8
R4 - non-compliance with remediation	0.75(0.89)	0.75(0.89)	−0.8/>0.4
R5 - stress	0.88(0.64)	0.88(0.64)	−2.3/0.020

### Outcome Measures

The effect of MCT was evaluated by assessing at baseline pre-treatment (T1, September 2009) and after treatment (T2, March 2010) calculating changes in the rating scales outlined above (T2-T1).

### Statistics

Statistics were calculated using SPSS-18. The distributions of the group scores were tested for statistically significant differences with asymptotic 2-tailed probability. Where groups were compared for changes in variables, the mean change was adjusted for variations between individuals in baseline levels using univariate analysis of variance.

## Results

## Main results

Table 4 shows that Mac-CAT-T Understanding increased after MCT when compared with the waiting list comparison group (p = 0.009), and GAF also significantly increased after MCT when compared to the waiting list group (p = 0.012).

**Table 4:** Measurements for MCT and waiting list comparison group compared before treatment (T1, September 2009), after treatment or equivalent delay for waiting list patients (T2, March 2010) and differences

	T1 (baseline)			T2 (after treatment or waiting list)			T2-T1 differences		
	No MCT	MCT	T/p	No MCT	MCT	T/p	No MCT	MCT	T/p
Mac-CAT-T									
Understanding	4.2(1.3)	4.7(1.2)	−0.9/>0.3	3.6(1.1)	5.1(0.9)	−3.1/0.008	−0.5(0.5)	+0.5(0.9)	−3.0/0.009
Reasoning	5.0(1.9)	5.5(2.0)	−0.5/>0.5	4.1(1.9)	6.2(1.1)	−2.6/0.023	−0.9(2.2)	+0.9(2.1)	−1.7/>0.1
Appreciation	2.5(1.6)	2.7(1.5)	−0.3/>0.7	2.4(1.5)	2.6(1.5)	−0.4/>0.7	−0.13(0.9)	−0.1(1.6)	−0.01/>0.9
Total	11.8(4.3)	12.9(4.1)	−0.6/>0.5	10.3(3.9)	13.9(2.5)	−2.3/0.041	−1.5(2.9)	+1.3(3.9)	−1.7/>0.1
Mac-CAT-FP									
Understanding	9.8(3.8)	9.8(3.5)	−0.04/>0.9	10.6(3.7)	10.6(2.6)	−0.7/>0.3	0.9(2.0)	2.2(1.7)	−1.5/>0.1
Reasoning	7.1(1.9)	7.4(1.6)	−0.3/>0.7	5.9(2.1)	7.8(2.2)	−1.95/>0.05	−1.3(1.9)	0.5(1.6)	−1.9/>0.05
Appreciation	8.3(3.5)	8.6(2.7)	−0.03/>0.7	9.0(2.4)	9.4(2.7)	−0.3/>0.7	0.8(2.7)	0.7(1.6)	0.02/>0.9
Total	23.9(7.5)	25.5(6.5)	−0.5/>0.6	25.1(7.0)	29.2(7.1)	−1.2/>0.2	1.3(5.9)	3.6(5.1)	−0.9/>0.3
GAF	54.6(8.7)	50.6(9.5)	0.9/>0.3	48.0(6.9)	57.2(9.8)	−2.6/0.021	−6.6(8.7)	6.6(12.0)	−2.8/0.012
PANSS									
PANSS positive	14.0(6.3)	11.4(3.7)	1.1/>0.2	15.3(5.4)	13.5(4.4)	0.8/>0.4	1.3(3.9)	2.2(4.9)	0.5/>0.6
PANSS negative	17.5(4.7)	17.7(6.7)	0.1/>0.9	20.5(4.2)	16.6(6.5)	0.6/>0.1	3.0(3.8)	−1.1(6.5)	1.7/>0.1
PANSS general	27.0(6.6)	31.7(8.6)	−1.4/>0.2	30.0(5.3)	29.4(10.2)	0.2/>0.8	3.0(4.9)	−2.4(8.3)	1.8/>0.1
PANSS total	58.8(14.9)	60.7(15.2)	−0.3/>0.7	66.0(8.3)	59.5(18.0)	1.0/>0.3	7.3(10.5)	−1.2(18.6)	1.3/>0.2

Means (standard deviations), T statistic with equal variances not assumed, p values 2-tailed.

Table 5 shows that after treatment, patients who attended group MCT did not have significant differences in the PANSS compared with the waiting list comparison group. After adjustment for baseline, those undergoing MCT had an improvement in the GAF compared to the waiting list group (p = 0.024). 

**Table 5:** Marginal means - changes in the Positive and Negative Symptoms of Schizophrenia (PANNS) and General Assessment of Function (GAF) in MCT group and waiting list group

	**MCT patients (n = 11)difference in marginal means (SEM) T2-T1**	**Waiting List group, (n = 8)difference in marginal means (SEM) T2-T1**	**Difference between treatment and waiting list group marginal means (SEM)**	**Significance, p (significant p value of < or = 0.05)**
**PANNS positive**	1.7 (1.3)	1.9 (1.5)	0.23(2.0)	>0.9
**PANNS negative**	−1.0 (1.5)	2.9 (1.7)	3.9 (2.3)	>0.05
**PANNS general**	−0.7 (2.1)	2.1 (2.5)	3.8 (3.4)	>0.2
**PANNS total**	−0.7 (4.1)	6.6 (4.8)	7.3 (6.4)	>0.2
**GAF**	5.4 (2.6)	−4.9 (3.1)	−10.3 (4.1)	0.024

Marginal means (SEM). Differences in marginal means have been adjusted for baseline levels.

Correlating changes in outcome measures (T2-T1) with the number of treatment sessions attended for all 19 patients including the waiting list comparison group, for MacCAT-T change in understanding score, T2-T1, Spearman r = +0.644 p = 0.004, reasoning change, r = +0.540, p = 0.021, appreciation change r = +0.284 p > 0.3, change in total MacCAT-T score r = +0.556, p = 0.016; for MacCAT-FP change in understanding score r = +0.250, p > 0.3, reasoning r = +0.410 p > 0.05, appreciation r = +0.159 p > 0.5, total MacCAT-FP score r = 0.236 p > 0.3. The number of treatment sessions attended also correlated with the improvement in GAF score (Spearman r = +0.592, p = 0.008) but not with change in any of the PANSS scales.

### Other Analyses

The data suggested a relationship between the baseline measure and the size of potential change. This was tested by Spearman correlation coefficient for all 19 patients, comparing each baseline measure with the change in that measure (T2-T1). For MacCAT-T understanding r = −0.185 p > 0.4, reasoning −0.717 p < 0.001, appreciation r = −0.427 p > 0.7 and for the MacCAT-T total score r = −0.467 p = 0.05. This confirms an expected inverse relationship between magnitude of baseline score and magnitude of change between the two time periods. Those with lower scores at baseline had greater increases. When only those who had treatment were considered, the correlations between baseline and change were greater.

Because of the relationship between the magnitude of baseline score and magnitude of change (T2-T1), to test the effect of treatment on MacCAT-T and MacCAT-FP measures, we used a general linear model to perform univariate analysis in which the difference for each parameter between baseline and post treatment measures was the dependent variable, treatment vs waiting list status was a fixed factor and the baseline measurement for that variable was the only covariate. Table 5 shows that there were no significant changes in PANSS score but GAF score improved after MCT when compared to the waiting list comparison group even when adjusted for baseline differences (p = 0.024). Table 6 further shows this improvement in the 'raw' GAF scores (p = 0.035). Patients who attended MCT demonstrated a change of +6.6 (SD 12.0) points on the GAF while patients who did not attend showed a change in GAF of −6.6 (SD 8.7) (ANOVA =7.0, df = 1, p = 0.017).

**Table 6:** Pre and post MCT GAF scores: 'raw' (unadjusted) data

	**MCT** n = 11	**Waiting list comparison group** n = 8	**ANOVA** f/p df = 1
T1: Baseline GAF	50.6 (9.5)	54.6 (8.8)	0.9/>0.3
T2: Post-MCT or waiting list GAF	57.2 (9.8)	48.0 (6.9)	5.1/0.035

Table 7 shows that after adjustment for baseline values there were improvements in Mac-CAT-T understanding (p = 0.011) and reasoning (p = 0.008) but not appreciation (p > 0.8), while the total Mac-CAT-T score also improved significantly (p = 0.019).

**Table 7:** Marginal means - changes in the MacArthur Competence Assessment Tool- Treatment (MacCAT-T) in patients attending MCT and waiting list comparison group when adjusted for baseline scores

	**MCT patients (n = 11)difference in marginal means (SEM) T2-T1**	**Waiting List group, (n = 8)difference in marginal means (SEM) T2-T1**	**Difference between treatment and waiting list group marginal means (SEM)**	Significance (* = significant p value of < or = 0.05)
Mac Cat – T				
(Total)	+1.53 (0.86)	−1.88 (0.96)	−3.4 (1.29)	0.019*
Mac Cat – T				
(Understanding)	+0.44 (0.22)	−0.52 (0.25)	−0.96(0.34)	0.011*
Mac Cat – T				
(Reasoning)	+ 1.08 (0.47)	−1.10 (0.53)	−2.18(0.71)	0.008*
Mac Cat – T				
(Appreciation)	−0.06 (0.40)	−0.17 (0.43)	−0.10(0.60)	>0.8

Table 8 shows that using the same statistical analysis for the MacCAT-FP, after adjustment for baseline scores there was a small improvement in the reasoning component of the MacCAT-FP (p = 0.049) but no significant differences in the other parameters assessed.

**Table 8:** Marginal means - changes in the MacArthur Competence Assessment Tool- Fitness to Plead (MacCAT-FP) in patients attending MCT and waiting list comparison group when adjusted for baseline scores

	**MCT patients (n = 11)difference in marginal means (SEM) T2-T1**	**Waiting List group, (n = 8)difference in marginal means (SEM) T2-T1**	**Difference between treatment and waiting list group marginal means (SEM)**	Significance (* = significant p value of < or = 0.05)
Mac CAT – FP				
(Total)	3.8 (1.6)	0.9 (1.9)	−2.9 (2.5)	>0.2
Mac CAT – FP				
(Understanding)	2.19 (0.49)	0.86 (0.57)	−1.33 (0.75)	>0.05*
Mac CAT – FP				
(Reasoning)	0.48 (0.54)	−1.29 (0.63)	1.77 (0.82)	0.049*
Mac CAT – FP				
(Appreciation)	0.74 (0.74)	0.73 (0.63)	0.010(0.98)	>0.9

## Discussion

## Key results

This was a small pilot study, a naturalistic prospective observational study allowing comparison of a treatment group and waiting list group. The patients in the study had been in-patients in a secure forensic hospital for almost four years, but still had positive symptoms of psychosis. We have shown that measures of mental capacity could be used as outcome measures for this treatment intervention and there were improvements in understanding and reasoning as measured by the MacCAT-T and reasoning as measured by the MacCAT-FP. PANSS scores did not improve but GAF scores did improve significantly when compared to the waiting list comparison group. The more sessions attended, the greater the improvement in GAF, Mac-CAT-T understanding, reasoning (but not appreciation) and total score, and in Mac-CAT-FP reasoning also.

Baseline measures correlated with magnitude of change. When adjusting for baseline scores, the changes were significantly greater for the MCT group compared to the waiting list group for Mac-CAT-T understanding, reasoning and total scores and Mac-CAT-FP reasoning as well as GAF. Metacognitive Training (MCT) may be used in patients with fairly severe psychotic illness, it is acceptable to patients and may be associated with benefit.

### Limitations

This was not a randomised controlled trial. We could not be certain that all assessments were blind to treatment status - the GAF and PANSS assessments were the most robust in this respect. Because the study size is small, we cannot be certain that some beneficial effects might have been missed for example concerning symptoms of psychosis, and it is possible that adverse effects could also have been missed (type II errors). In view of the small sample size, it is possible also that some of the differences found may be false positives (type I errors).

Bias in the results might have arisen from selection bias, whereby the more able were put forward for treatment ahead of others who were relegated to the waiting list. We could find no evidence of this in comparisons of relevant baseline measurements (Tables 1–2) though an RCT would provide greater reassurance. It remains possible that other, unmeasured confounding factors might account for the differences between treatment and waiting list groups. Information bias can arise due to misclassification of the level of exposure to the intervention in any study. We found that the number of sessions completed was correlated with the change in MacCAT-T understanding and reasoning but not appreciation. It also correlated with changes in GAF but not in MacCAT-FP scales or PANSS scales. While there may be many unknown and unmeasured confounding factors and the numbers in this study are small, the MacCAT-T corresponds in content with the content of the MCT programme, while the MacCAT-FP does not. This suggests that the number of sessions completed is a valid measure of exposure to the intervention in this study.

For service reasons we included patients with a range of impairments rather than selecting only the most able or least able. This imposes 'ceiling' and 'floor' effects on the potential for magnitude of change in outcome measures even when means do not differ significantly at baseline. Correction for the baseline was therefore necessary when comparing magnitude of change. This does not impose a statistical bias as long as the intervention occurs only after the baseline measurement and there is no evidence for a prior trend [41].

Observer bias may have arisen as it was not possible to blind the raters regarding interventions received and this may have influenced results. All these factors mean that it is not possible to draw strong conclusions about the effectiveness of the intervention.

### Interpretation

Moritz and Woodward [6,7] define MCT as a hybrid of psycho-education, cognitive remediation and cognitive-behavioural therapy. We suggest that there is some correspondence between these modalities and deficits in understanding, reasoning and appreciation.

### Psycho-education and 'Understanding'

Psycho-education has been shown to add a small-to-medium effect size to medication and is an especially viable strategy for patients who are medication resistant [4]. MCT aims to educate patients about paranoia and delusions and offers an opportunity for individual and group reflection on personal psychotic experiences. MCT thus aims to enhance patients’ ability to understand their symptoms of mental illness. The MacCAT-T assesses patients’ capacity to understand symptoms of their mental illness by giving them relevant information and assessing their ability to retain, retrieve and explain the information learnt. Changes in the MacCAT-T-Understanding scale could be viewed as a method of assessing the effectiveness of MCT in improving patients' knowledge and understanding of mental illness. The improvement in MacCAT-T 'Understanding' observed in this study suggests that positive changes occur for the patient in understanding symptoms of their illness such as paranoia and delusions over the course of MCT.

### 'Reasoning' and remediation

The improvements in reasoning as measured by both the Mac-CAT-T and Mac-CAT-FP 'Reasoning' sub-scales appear to represent a benefit that is more general, dealing with issues concerning the real world, relevant to the forensic patients assessed. Cognitive remediation can be explained as correction of a fault or deficiency in cognition [42]. Cognitive Remediation Therapy (CRT) generally concentrates on form rather than content of thought and the main outcome of therapy is to improve thinking and reasoning skills themselves. MCT aims to bring about cognitive remediation through components such as the 'jumping to conclusions' module. Attributional biases are also dealt with in MCT in a similar manner where participants are encouraged to find, evaluate and combine different possible explanations. This could be viewed as leading to improved reasoning skills as evaluated using the MacCAT-T and MacCAT-FP reasoning sub-scales. The improvements found in MacCAT-T and Mac-CAT-FP reasoning following MCT demonstrate that MCT has an effect on patients’ reasoning capacity that can be detected using these assessment tools.

### Congnitive-behaviour therapy, insight and 'Appreciation'

The lack of any effect on either Mac-CAT-T or Mac-CAT-FP 'Appreciation' - may represent a limit for this programme as presently constituted. Appreciation of a mental illness as relevant to one's self and the effect it can have on one's life was not changed significantly by MCT. This has been observed before to be the most resistant to change [16,35]. While 'appreciation' is generally taken as one of the components of mental capacity relevant to the assessment of functional capacities in legal contexts, it is defined as a narrower concept than 'insight', and may or may not be a sub-set of insight [43,44].

### Generalisability

Apart from the reasoning component of the MacCAT-FP, MCT had no impact on other aspects of fitness to plead such as knowledge (understanding) of the judicial system or an appreciation of how the judicial system can affect the individual and their lives. This was expected as it was not consistent with the aim or content of MCT. It is interesting that the only change seen in the MacCAT–FP was in reasoning which means that the reasoning skills or abilities enhanced in MCT were transferrable to other decision making scenarios where reasoning skills are required. Both functional capacity assessments (MacCAT–FP and MacCAT-T) measure reasoning by different methods and both showed that MCT was associated with an improvement on reasoning ability.

Reasoning, as measured in the MacCAT–T assesses consequential and comparative reasoning. It does this in the form of a structured discussion around treatment options for the patient's own illness. Investigators are encouraged to probe to explore the patient's explanations for their choices and reasoning process in arriving at their decisions. The Mac-CAT-T assesses if the patient is able to see consequences of different treatments and to compare different treatment options. It assesses if patients can generate practical consequences of their illness in their day-to-day lives and the positive and negative impact that medication, or no medication, might have on them. The MacCAT–T tool requires the person assessed to indicate a final treatment option and explores their reasoning around this final option. All of these components give a good indication of a patient's reasoning abilities.

The MacCAT-FP is a very interactive, enjoyable assessment tool that centres on presenting the patient with a fictional legal narrative. The reasoning component of this tool then adds to the story by presenting the patient with a series of pairs of facts then asking him/her which fact would be more important within the context of the story. A series of similar questions gives additional facts and the end result is a comprehensive evaluation of the patient's comparative and consequential reasoning abilities given these hypothetical scenarios. The methods of assessing reasoning in both the MacCAT-T and the MacCAT-FP are very different in form yet it appears that MCT had an effect on reasoning that went beyond the specific knowledge of what was learned in the MCT sessions.

There were no significant changes found in positive, negative or general symptoms of psychosis as measured by the PANSS. This is in keeping with previous studies for example [9] which found trends toward improvement in all subscales of the PANSS but these did not reach statistical significance. Moritz et al. [45] showed that individual and group MCT significantly improved a novel PANSS delusional sub-scale and a newer more sensitive measure [46] which may match with the aims of MCT better than the PANSS as used in this study and earlier studies. Recently, the use of newer outcome measures has shown improvements in the distress due to delusions, memory and social relationships [30]. Symptom improvement is not necessarily the best guide to functional improvement and other measures, such as functional mental capacity should have a place as outcome measures for such treatments.

In our study there was an improvement in the GAF score in patients who underwent MCT compared to the waiting list comparison group. This may be taken as evidence of practical benefits of MCT in the lives of patients, in keeping with improvements in practical decision-making capacities. It may be that such improvements are more important than improvements in symptoms.

In terms of the strengths of the study, this was the first study to attempt to evaluate MCT in a forensic setting. This study is also novel in that it attempts to use as outcome measures two different functional mental capacities and their component mental capacities of understanding, reasoning and appreciation for decision making following a focused psychological intervention. This study shows the acquisition of reasoning skills through MCT and their transferability to other contexts as discussed above. Most importantly, improvement in the GAF indicated that MCT may be associated with improvements in social and occupational functioning in general.

## Conclusions

This research was done as an audit for the service evaluation of MCT, a psychological intervention known to have benefits for symptoms of psychosis. We have shown that measures of mental capacity to consent to treatment are sufficiently sensitive to change to be used as outcome measures in a study such as this, even with small numbers and less than perfect study design. MCT was associated with improved knowledge of mental illness, generalised reasoning skills and global functioning of patients with a psychotic illness.

## Competing interests

The authors declare that they have no competing interests.

## Authors’ contributions

MN collected the data, rated the capacity assessments and coordinated the assessment project. AN organised and co-ordinated the MCT intervention. AN and ZA collated the PANSS and GAFs. MN and AN produced the first draft of the paper. MD and SO’D rated the Dundrum 1 assessment tool. HGK designed the pragmatic study and carried out the data analysis. All contributed to the authorship of the paper and approved the final manuscript.
